# Induction of protein body formation in plant leaves by elastin-like polypeptide fusions

**DOI:** 10.1186/1741-7007-7-48

**Published:** 2009-08-07

**Authors:** Andrew J Conley, Jussi J Joensuu, Rima Menassa, Jim E Brandle

**Affiliations:** 1 Department of Biology, University of Western Ontario, London, ON, Canada; 2 Southern Crop Protection and Food Research Centre, Agriculture and Agri- Food Canada, London, ON, Canada; 3 VTT Technical Research Centre of Finland, Espoo, Finland; 4 Vineland Research and Innovation Centre, Vineland Station, ON, Canada

## Abstract

**Background:**

Elastin-like polypeptides are synthetic biopolymers composed of a repeating pentapeptide 'VPGXG' sequence that are valuable for the simple non-chromatographic purification of recombinant proteins. In addition, elastin-like polypeptide fusions have been shown to enhance the accumulation of a range of different recombinant proteins in plants, thus addressing the major limitation of plant-based expression systems, which is a low production yield. This study's main objectives were to determine the general utility of elastin-like polypeptide protein fusions in various intracellular compartments and to elucidate elastin-like polypeptide's mechanism of action for increasing recombinant protein accumulation in the endoplasmic reticulum of plants.

**Results:**

The effect of elastin-like polypeptide fusions on the accumulation of green fluorescent protein targeted to the cytoplasm, chloroplasts, apoplast, and endoplasmic reticulum was evaluated. The endoplasmic reticulum was the only intracellular compartment in which an elastin-like polypeptide tag was shown to significantly enhance recombinant protein accumulation. Interestingly, endoplasmic reticulum-targeted elastin-like polypeptide fusions induced the formation of a novel type of protein body, which may be responsible for elastin-like polypeptide's positive effect on recombinant protein accumulation by excluding the heterologous protein from normal physiological turnover. Although expressed in the leaves of plants, these novel protein bodies appeared similar in size and morphology to the prolamin-based protein bodies naturally found in plant seeds. The elastin-like polypeptide-induced protein bodies were highly mobile organelles, exhibiting various dynamic patterns of movement throughout the cells, which were dependent on intact actin microfilaments and a functional actomyosin motility system.

**Conclusion:**

An endoplasmic reticulum-targeted elastin-like polypeptide fusion approach provides an effective strategy for depositing large amounts of concentrated heterologous protein within the limited space of the cell via storage in stable protein bodies. Furthermore, encapsulation of recombinant proteins into physiologically inert organelles can function to insulate the protein from normal cellular mechanisms, thus limiting unnecessary stress to the host cell. Since elastin-like polypeptide is a mammalian-derived protein, this study demonstrates that plant seed-specific factors are not required for the formation of protein bodies in vegetative plant tissues, suggesting that the endoplasmic reticulum possesses an intrinsic ability to form protein body-like accretions in eukaryotic cells when overexpressing particular proteins.

## Background

Seeds provide an attractive alternative to conventional large-scale recombinant protein expression systems since they can produce relatively high heterologous protein yields in a stable, compact environment for long periods of time, assisting in storage, handling, and transport of the transgenic product [1]. Compared with other eukaryotes, plants are unique in their ability to naturally store large reservoirs of protein in specialized endoplasmic reticulum (ER)-derived compartments in developing seeds [2].

Prolamins are the most predominant class of seed storage proteins found in most cereals, such as maize, rice, and wheat [3]. In general, prolamins contain proline-rich domains and are alcohol-soluble, reflecting their general hydrophobic nature [4]. γ-Zein, a prolamin and the major constituent of maize storage proteins, contains a highly repetitive sequence (PPPVHL)_8 _that adopts an amphipathic helical conformation, which is able to self-assemble and may be responsible for this protein's ability to be retained in the ER, despite the absence of an H/KDEL ER-localization signal [5,6]. Although the sequestration mechanisms are not well understood, prolamin seed storage proteins are synthesized on the rough ER and deposited as large, dense accretions known as protein bodies (PBs) [7,8].

Although plant seeds have many positive attributes, major hurdles still exist for using seed-based systems as recombinant protein bioreactors. For example, there is a strong reluctance among scientists, regulators, and the general public to use seeds of major crops (that is, maize, rice and wheat) for biopharmaceutical production, given the possibility of contaminating the food chain [9]. In addition, potential environmental damage could result from the dispersal of transgenes into the environment through pollen or seed [10]. Alternatively, tobacco is well-suited as a production system for recombinant proteins since it has a high biomass yield and is readily amenable to genetic engineering. Because the tobacco expression platform is based on leaves, harvesting occurs prior to flowering, thus minimizing the possibility of gene leakage into the environment. Most importantly, tobacco is a non-food, non-feed crop, which minimizes regulatory barriers by eliminating the risk of plant-made recombinant proteins entering the food supply [11,12]. However, the low production yields of many recombinant proteins in tobacco remains a serious problem for this host system, since foreign proteins are often unstable and particularly susceptible to proteolytic degradation in the aqueous environment of leafy crops [13,14].

A useful strategy for increasing the accumulation of recombinant proteins in plants may be to integrate the components responsible for stable seed protein storage with the inherently biosafe and high biomass-yielding leaf-based tobacco expression platform. In fact, it has recently been shown that prolamin storage proteins are capable of enhancing the accumulation of recombinant proteins in vegetative leaf tissues based on their ability to induce the formation of ER-derived PBs [15-18]. Furthermore, the induced PBs are dense organelles which can facilitate the recovery and purification of fused recombinant proteins by simple and inexpensive density-based separation methods [15,19].

Elastin-like polypeptides (ELPs) are synthetic biopolymers composed of a repeating pentapeptide 'VPGXG' sequence which occur in all mammalian elastin proteins [20]. In an aqueous solution, ELPs undergo a reversible inverse phase transition from soluble protein into insoluble hydrophobic aggregates that form β-spiral structures when heated above their transition temperature (*T*_*t*_) [21,22]. This thermally-responsive property of ELP is also transferred to fusion partners, enabling a simple, rapid, scalable, and inexpensive non-chromatographic method for protein purification called 'inverse transition cycling' (ITC) [23]. ITC has been used to purify cytokines [24,25], antibodies [26], and spider silk proteins [27] from transgenic plants. As an additional beneficial side-effect, ELP fusions have also been shown to significantly enhance the accumulation of a range of different recombinant proteins in transgenic tobacco leaves [28,29] and seeds [30]. Although it is thought that ELP tags confer increased stability or solubility to their fusion partner, the means by which ELP increases the production yield of recombinant proteins *in planta *has not yet been established.

The biochemical properties of ELPs and prolamins, such as γ-zein, share many similarities. For example, both proteins are generally hydrophobic and proline-rich, with the ability to self-assemble and form supramolecular secondary structures consisting of helices and spirals as a result of their highly repetitive sequences [6,31]. These shared characteristics led us to hypothesize that ELP fusions may increase heterologous protein yield in a manner analogous to prolamin seed storage proteins. The objective of this study was to elucidate the mechanism by which ELP increases recombinant protein accumulation in the ER of plants and to determine the utility of ELP in various intracellular compartments. Thus, green fluorescent protein (GFP)-ELP fusions were targeted to the cytoplasm, chloroplasts, apoplast, and the ER. Interestingly, we found that GFP-ELP fusions targeted to the ER tended to form novel PB-like structures in leaves, which may exclude the heterologous protein from normal physiological turnover and may be responsible for ELP's positive effect on recombinant protein accumulation.

## Results

### Localization of elastin-like polypeptide fusions to the cytoplasm, chloroplasts, and apoplast

**Figure 1:**
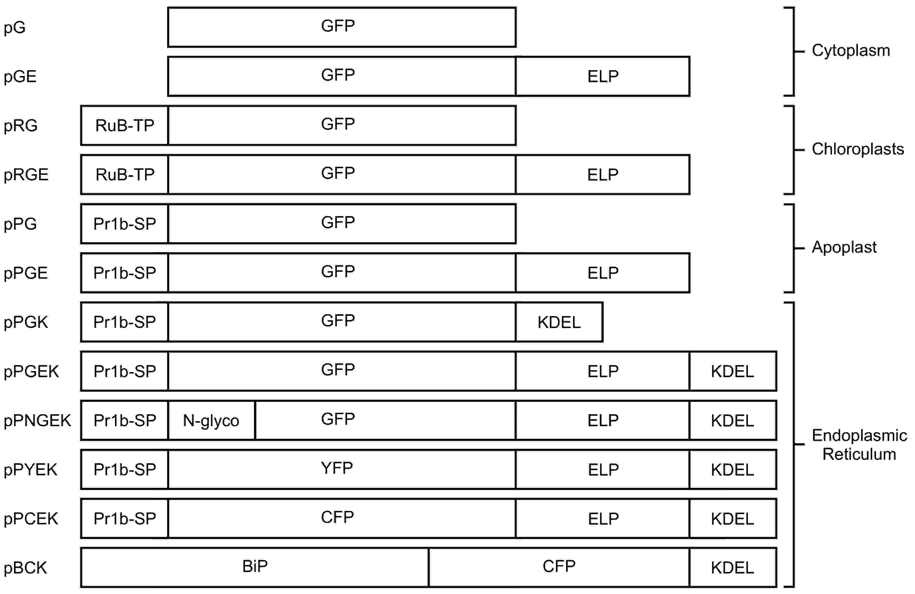
**Schematic representation of the genetic constructs used for *Agrobacterium*-mediated transient expression in *Nicotiana benthamiana *leaves**. All transgene expression fragments were placed under the control of the cauliflower mosaic virus 35S promoter, a tCUP 5'-untranslated region and the nopaline synthase terminator. RuB-TP, transit peptide from the tobacco small subunit RuBisCo gene; Pr1b-SP, tobacco secretory signal peptide; N-glyco, *N*-glycosylation motif (GELVSNGTVT); BiP, tobacco immunoglobulin heavy chain binding protein; GFP, green fluorescent protein; YFP, yellow fluorescent protein; CFP, cyan fluorescent protein; ELP, elastin-like polypeptide tag (28×VPGVG); KDEL, endoplasmic reticulum retention signal.

To better understand the mechanisms that allow ELP fusions to enhance recombinant protein accumulation in plants, the effect of ELP on the subcellular localization of GFP was investigated. Plant expression vectors were constructed to produce GFP-tagged ELP protein fusions targeted to the cytoplasm, chloroplasts, apoplast, and the ER, along with non-fused GFP controls (Figure 1). All coding sequences were introduced into a plant binary vector, with transcription driven by the constitutive CaMV 35S promoter. The GFP constructs were transiently expressed in leaves via agro-infiltration and the subcellular localization was analyzed by confocal laser scanning microscopy.

**Figure 2:**
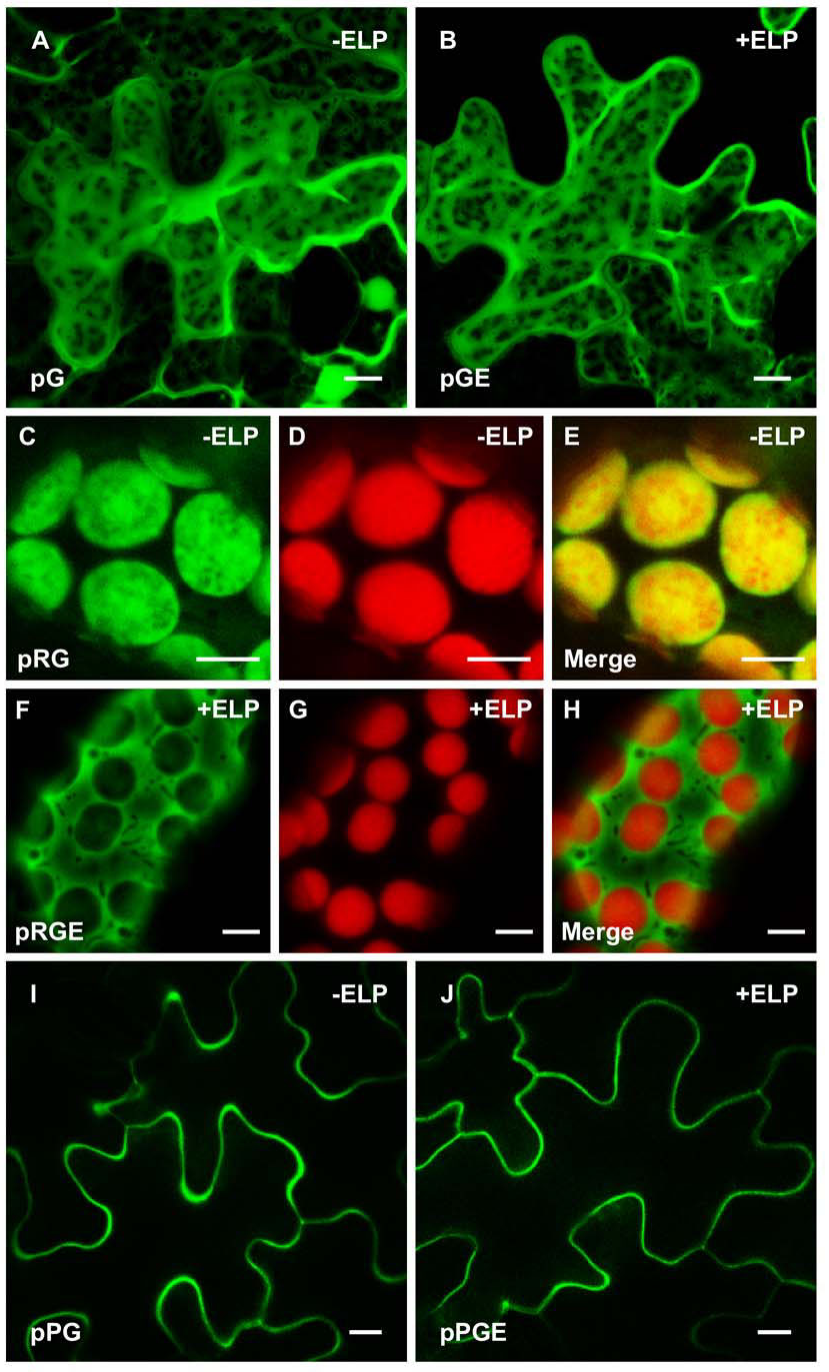
**Subcellular localization of green fluorescent protein and green fluorescent protein-elastin-like polypeptide targeted to the cytoplasm, chloroplasts and apoplast**. The localization constructs were agro-infiltrated into *Nicotiana benthamiana *leaves and visualized by confocal microscopy. The pG **(A) **and pGE **(B) **proteins were both visible as diffuse expression surrounding variously shaped and sized organelles throughout the cell. Green fluorescent protein (GFP) fluorescence was most concentrated in the cytoplasmic strands and the nucleus. In mesophyll cells expressing chloroplast-targeted GFP (pRG), the GFP fluorescence was localized to the chloroplasts **(C)**, which was confirmed by also detecting the chlorophyll autofluorescence **(D)**. **(E) **Merged image of (C) and (D) showing complete co-localization of pRG and the chloroplasts. In the presence of an elastin-like polypeptide (ELP) fusion tag, the chloroplast-targeted GFP (pRGE) appeared to accumulate in the cytoplasm **(F) **and was excluded from the chloroplasts **(G)**. **(H) **Merged image of (F) and (G), demonstrating that an ELP fusion tag prevents the accumulation of GFP in the chloroplasts. For both pPG **(I) **and pPGE **(J)**, the images were taken from a cross-section of the cells showing a secreted pattern of fluorescence consistent with apoplast localization. Bar, 10 μm (A, B, I, J); 5 μm (C to H).

In the absence of additional targeting sequences, the cytoplasmic-targeted proteins (pG and pGE) showed the same pattern of localization and were visible as diffuse expression across the entire cell (Figure 2A and 2B). In both cases, the GFP fluorescence surrounded variously shaped and sized organelles and accumulated in the nucleus as a result of GFP's relatively small size, which allows for passive diffusion through the nuclear pores [32,33]. To direct the protein into the chloroplasts, the transit peptide from the tobacco small subunit RuBisCo gene was fused to GFP, in the absence or presence of an ELP tag. Without an ELP tag, the GFP control protein was appropriately localized to the chloroplasts, which was verified by overlaying its image with the chlorophyll autofluorescence (Figure 2C to 2E). On the other hand, when ELP was fused to chloroplast-targeted GFP, the fluorescence appeared to be excluded from the chloroplasts and subsequently occupied the cytoplasmic space surrounding the chloroplasts (Figure 2F to 2H). As expected, the secreted forms of GFP, with or without an ELP tag, were observed to accumulate in the apoplast since they contained a tobacco secretory signal peptide without additional targeting sequences (Figure 2I and 2J).

### Accumulation of elastin-like polypeptide fusions in various subcellular compartments

**Figure 3:**
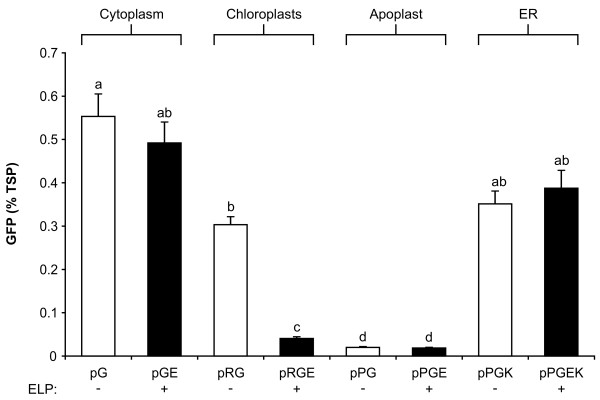
**Accumulation of green fluorescent protein in various subcellular compartments, in the presence or absence of an elastin-like polypeptide tag**. The concentration of green fluorescent protein (GFP) was measured by quantitative fluorometric analysis from leaf sectors harvested from *Nicotiana benthamiana *plants 4 days post-agro-infiltration. Each column represents the mean value (*n *= 8), and the standard error of the mean is represented with error bars. Columns not connected with the same letter are significantly different (*P *< 0.05) from each other using Tamhane's T2 test. TSP, total soluble protein.

To evaluate the general utility of ELP fusion tags in various subcellular compartments in plants, the expression constructs were agro-infiltrated into *Nicotiana benthamiana *leaves and the concentration of GFP was quantified by measuring the fluorescence intensity of the leaf extracts. Of the four subcellular compartments tested, GFP accumulated to the highest level (0.55% of total soluble protein (TSP)) in the cytoplasm, followed by the ER, chloroplasts, and apoplast (Figure 3). The presence of an ELP fusion tag had a negligible effect on the concentration of GFP in the cytoplasm, apoplast, or ER. We speculate that in contrast to the less-stable recombinant proteins investigated in prior studies [26,28-30], ELP did not increase the GFP yield in *N. benthamiana *leaves because GFP is already a highly stable and soluble protein [34-36]. For the chloroplast-targeted construct, the addition of an ELP tag significantly decreased the concentration of GFP eight-fold, which agrees with the confocal analysis showing no observable accumulation of the fusion protein in the chloroplasts (Figure 2F to 2H). The ER-retained constructs, with or without an ELP tag, produced approximately 20 times more GFP than their fully secreted apoplastic counterparts, suggesting that the ER provides a better environment within the secretory pathway for the accumulation of GFP or GFP-ELP. To validate the quantitative results obtained in *N. benthamiana*, all of the expression constructs were also agro-infiltrated into *Nicotiana tabacum *leaves. Irrespective of the *Nicotiana *plant host, the expression patterns observed between the constructs were very similar, with comparable amounts of GFP produced for each construct (data not shown). Clearly, the subcellular location of GFP greatly affected its accumulation, with the cytoplasm and ER being the best of the compartments tested.

### Hyperexpression of an endoplasmic reticulum-targeted elastin-like polypeptide fusion induces the formation of protein bodies

**Figure 4:**
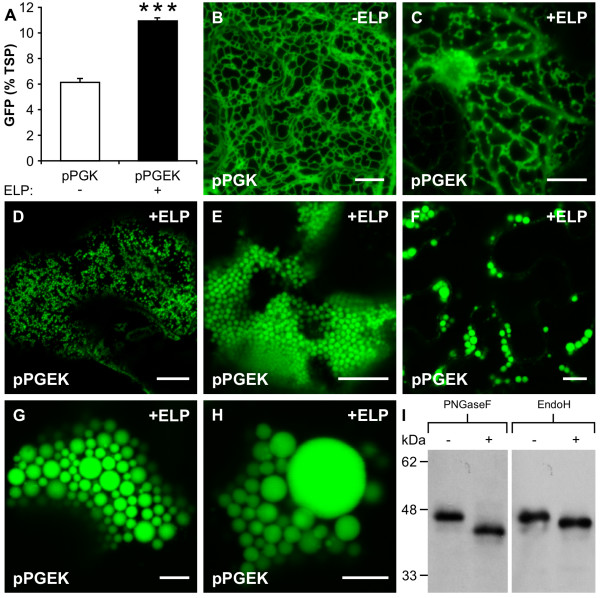
**Hyperexpression of an endoplasmic reticulum-targeted elastin-like polypeptide fusion induces the formation of protein bodies in leaves**. **(A) **Accumulation of endoplasmic reticulum- (ER-)targeted green fluorescent protein (GFP), with or without an elastin-like polypeptide (ELP) tag, when transiently co-expressed with the p19 suppressor of gene silencing in the leaves (*n *= 8) of *Nicotiana benthamiana *plants. ***, significant difference (*P *< 0.001). **(B) **Confocal image of the ER-targeted GFP control protein (pPGK) demonstrating the open polygonal network consistent with ER-localization. **(C-H) **In the presence of an ELP fusion tag, the ER-targeted GFP (pPGEK) was detected in brightly fluorescing spherical-shaped particles distributed throughout the cells of the leaf. (C) The novel PBs were closely associated with the ER tubules as small punctuate structures early on in the PB-formation process. (D) With time, the PB-like organelles continued to grow and appeared to be released from the ER into the cytoplasm, where they remained. (E-H) The PBs obtained various sizes and tended to cluster together within the cell, although the distribution pattern was quite variable. The majority of PBs had an observable diameter of between 0.5 and 1.0 μm, but larger PBs were seen at lower frequencies with some approaching diameters of 8.0 μm. **(I) **Deglycosylation of an ER-targeted GFP-ELP fusion engineered to contain an *N*-glycosylation motif (GELVSNGTVT). Total protein extracts (3 μg/lane) from agro-infiltrated plant tissue expressing pPNGEK were incubated for 24 h in the presence (+) or absence (-) of peptide *N*-glycosidase F (PNGaseF) or endoglycosidase H (EndoH) and then subjected to sodium dodecylsulphate-polyacrylamide gel electrophoresis and immunoblotted with an anti-GFP antibody. Bar, 10 μm (B-F); 5 μm (G, H).

Previous studies have demonstrated that ELP fusion tags have the ability to significantly enhance the accumulation of various ER-targeted recombinant proteins in plants [28-30]. Moreover, ELP has been shown to promote the formation of distinct intracellular organelles within the leaves of *N. tabacum *(unpublished data). To better establish the role of ELP tags in the accumulation of heterologous proteins, *N. benthamiana *plants were agro-infiltrated with the ER-targeted constructs (pPGK and pPGEK) along with the p19 suppressor of gene silencing, which has been found to significantly increase the production levels of recombinant proteins in plants [37-39]. In the presence of p19, the levels of pPGK and pPGEK were enhanced by approximately 20- and 30-fold respectively. In the *N. benthamiana *leaf extracts, the accumulation of GFP reached 11% of TSP with the ELP tag, which was almost two times higher than the same construct without an ELP tag (Figure 4A).

The fluorescence of the ER-targeted GFP control protein (pPGK) resembled a characteristic reticulate pattern (Figure 4B) consistent with the normal ER morphology of plant epidermal cells [40]. Relative to the control protein (pPGK), the distribution pattern of GFP was very different in the presence of an ELP fusion tag (pPGEK). The ER-targeted GFP-ELP fusion was easily observed as brightly fluorescing spherical-shaped structures distributed throughout the cells of the leaf following agro-infiltration (Figure 4C to 4H). The spherical particles, which are assumed to be PBs, were highly abundant in the vast majority of *N. benthamiana *leaf cells following hyperexpression of the pPGEK protein. The same pPGEK-expressing cells also showed the network pattern of the ER; however, the fluorescence intensity within the ER was fainter than the densely-packed, very bright spherical structures, making it difficult to image both simultaneously.

In general, both the size and number of PBs tended to increase with time following agro-infiltration. When the level of GFP fluorescence was relatively low, the PBs could be visualized concurrently with the ER network (that is, 1 to 2 days post-agro-infiltration). The PBs appeared to originate at the ER as small punctuate structures (Figure 4C). Over time (that is, 3 to 6 days post-agro-infiltration), the accumulation of GFP continued to increase and the PBs appeared to be excreted from the ER into the cytosol, where they remained as cytoplasmic organelles (Figure 4D). A collection of confocal images were compiled together to create a three-dimensional rendering of the PBs [see Movie S1 in Additional file 1]. Although the distribution pattern of the PBs was highly variable, they were most often found clustered together within the cell (Figure 4E to 4H) [see Movies S2 and S3 in Additional files 2 and 3]. Typically, the spherical PBs had an observable diameter of between 0.5 and 1.0 μm (Figure 4D and 4E). However, the size of the novel PBs was fairly heterogeneous (Figure 4F to 4H), with some of the PBs having diameters of 8.0 μm (Figure 4H), approaching the size of the cell's nucleus.

Surprisingly, small PBs (<1.0 μm in diameter) were also occasionally observed in the cells when expressing the unfused GFP control protein (pPGK) in the absence of p19. When quantified, only 5% of the cells expressing pPGK (*n *= 500) exhibited the presence of small PBs, whereas over 50% of the cells expressing pPGEK (*n *= 500) demonstrated the presence of small or large (>1.0 μm in diameter) PBs (Table 1). In both cases, the remaining cells showed an ER-like pattern of fluorescence. These results demonstrate that the addition of an ELP fusion tag was responsible for significantly enhancing the formation of the spherical PB-like organelles; however, GFP alone may have a propensity to aggregate into PBs when expressed at high levels inside the ER. When pPGK or pPGEK were hyperexpressed in the ER in the presence of p19, both the frequency and size of PBs observed in the plant cells were drastically increased. Approximately 44% of the cells (*n *= 500) expressing pPGK contained PBs, whereas almost all the cells (96%, *n *= 500) expressing pPGEK contained PBs (Table 1). When expressing these proteins at higher levels, the size distribution of the PBs was significantly shifted towards larger PBs. Most notably, the presence of an ELP tag (pPGEK) enhanced the occurrence of large PBs six-fold, relative to the unfused control (pPGK). These results support the notion that ELP plays an important, although not fully understood, role in the formation of PBs in plant cells expressing large quantities of recombinant proteins. Throughout the analyses, it was consistently observed that the most brightly fluorescing cells were also the cells possessing the highest number of PBs, typically of the large variety.

**Table 1:** Quantification of the transformed cells containing protein bodies and their respective size distribution

Experimental Condition^a^		Protein bodies		Protein body size	
			
		Absence^b ^(%)	Presence (%)	Small^c ^(%)	Large^d ^(%)
pPGK	(-p19)	95.0	5.0	5.0	0.0
pPGEK	(-p19)	49.8	50.2	46.4	3.8
					
pPGK	(+p19)	56.4	43.6	30.4	13.2
pPGEK	(+p19)	4.4	95.6	17.2	78.4

^a^For each experimental condition, a single leaf was agro-infiltrated on 10 different *Nicotiana benthamiana *plants and visualized by confocal microscopy 4 days post-transfection. For each of the 10 individually infiltrated leaves, five 7-mm leaf discs were excised and 10 cells were analyzed at random from each leaf disc to avoid any bias. Thus, 500 individual cells were classified for the presence or absence of protein bodies (PBs) and for the maximum size of PBs observed within each cell.

^b^These cells exhibited an endoplasmic reticulum-like pattern of fluorescence.

^c^PB diameter was <1.0 μm.

^d^PB diameter was >1.0 μm. These cells also contain small PBs.

Even though the pPGEK construct contains a KDEL ER-retention signal, we wanted to ensure that the PBs were ER-derived and not the result of incorrect protein trafficking to later stages of the secretory pathway. Thus, the N-terminus of the pPGEK coding sequence was modified to incorporate a potential *N*-glycosylation motif (that is, GELVSNGTVT), in order to test whether the protein was being transported through the Golgi stack by diagnostic glycosidase treatment [41]. The resulting protein, pPNGEK, retained the same pattern of GFP localization as the pPGEK construct, consisting of bright fluorescence in the ER and PBs (data not shown). To characterize the glycosylation of pPNGEK, transgenic plant extracts were treated with various glycosidases, followed by Western blot analysis. The pPNGEK protein was fully susceptible to digestion by peptide *N*-glycosidase F (PNGaseF) and endoglycosidase H (EndoH), resulting in mobility shifts for the protein (Figure 4I), which is consistent with the glycosylation pattern of ER-retained plant glycoproteins. Taken together, these results demonstrate that the KDEL-tagged pPNGEK protein was sensitive to PNGaseF and EndoH, suggesting a high mannose oligosaccharide structure indicative of ER localization with efficient retention/retrieval from the *cis*-Golgi. Furthermore, no colocalization was observed when pPGEK was transiently expressed along with a sialyltransferase signal anchor sequence [42] fused to red fluorescent protein, which specifically labeled the plant's Golgi bodies (data not shown).

### Movement of the novel protein bodies is highly dynamic and dependent on the actomyosin motility system

The PBs resulting from ER-targeted GFP-ELP (pPGEK) expression were observed to be highly mobile organelles, therefore time-lapse confocal images were taken of the fluorescent bodies in the epidermal leaf cells of *N. benthamiana*. We believe that this is the first reported example of PB mobility. The PBs exhibited various patterns of movement throughout the plant cells [see Movies S4 to S6 in Additional files 4, 5 and 6], which is very reminiscent of Golgi stack trafficking. For example, the PBs generally moved in a stop-and-go manner, alternating between periods of rapid vectorial movement and periods of relatively static, non-directed oscillation resembling Brownian motion. Most often, the PBs moved throughout the cell in a sporadic, saltatory fashion, but they could also be rapidly transported in a unidirectional manner via cytoplasmic streaming [41,43,44]. Thus far, the significance of incessant PB movement in the plant cells is unclear, as they appear to move continuously about the cell without a final destination.

**Figure 5:**
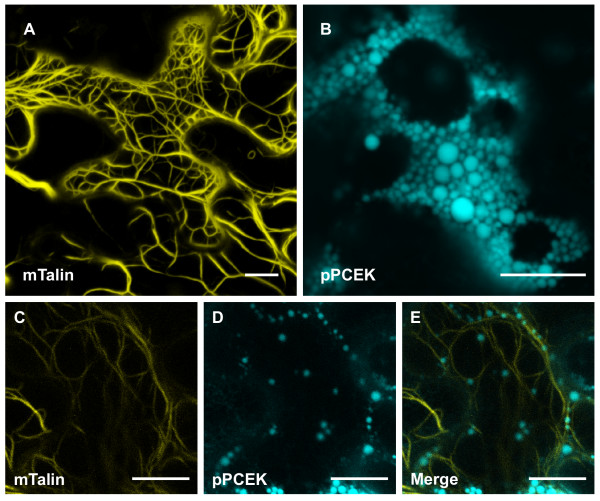
**Co-alignment of protein bodies with actin microfilaments**. **(A) **Expression of the mouse talin (mTalin) actin-binding domain fused with YFP localizes to the actin cables of the cytoskeleton within leaf epidermal cells after agro-infiltration. **(B) **An endoplasmic reticulum-targeted cyan fluorescent protein-elastin-like polypeptide fusion (pPCEK) accumulated as protein bodies (PBs) within the cell's cytoplasm. **(C-E) **When co-expressed, the induced novel PBs co-aligned with the actin microfilaments. Bar, 10 μm.

In plants, trafficking of organelles, such as Golgi bodies, mitochondria, and peroxisomes, occurs via the actomyosin motility system, which is empowered by myosin motors and is dependent on the actin cytoskeleton framework coextensive with the ER [43,45]. To date, the movement of PBs and the mechanism responsible for their transport have not been investigated. To explore the possibility that PBs are associated with the actin cytoskeleton, co-localization of an mTalin-YFP fusion [46], which serves as a reporter for the microfilaments, and an ER-targeted CFP-ELP fusion (pPCEK), was performed. Expression of the mTalin-YFP construct revealed a filamentous network (Figure 5A) resembling the actin cytoskeleton of plant cells [42,47,48]. Replacing GFP (pPGEK) with CFP (pPCEK) in the ER-targeted ELP fusion resulted in the same localization pattern, consisting of fluorescence emitting from the ER and PBs (Figure 5B). Co-expression of mTalin-YFP with pPCEK showed that most of the PBs co-aligned with the microfilaments (Figure 5C to 5E).

Since the PBs were observed to be associated with the actin cytoskeleton, time-lapse imaging experiments were performed to determine the dynamic status of the PBs following treatment with latrunculin B, which is an inhibitor of actin polymerization. Disruption of the cytoskeleton blocked intracellular trafficking of the PBs [see Movie S7 in Additional file 7], indicating that normal translational movement of PBs is dependent on intact microfilaments. Recently, myosin XI-K has been shown to play a major role in the movement of subcellular organelles [45,49]. Overexpression of a dominant-negative mutant of myosin XI-K prevents nearly all Golgi trafficking [45], so the mutant myosin XI-K tail was co-expressed with pPGEK to determine its effect on PB movement. In the presence of the myosin XI-K tail, PBs were still able to form in the cells, but their movement was severely halted [see Movie S8 in Additional file 8], suggesting that myosin XI-K is required for the trafficking of PBs. Taken together, these results demonstrate that PB movement is dependent upon the integrity of actin microfilaments and a functional actomyosin motility system.

### Endoplasmic reticulum luminal binding protein is localized to the protein bodies, but does not specifically interact with the elastin-like polypeptide tag

**Figure 6:**
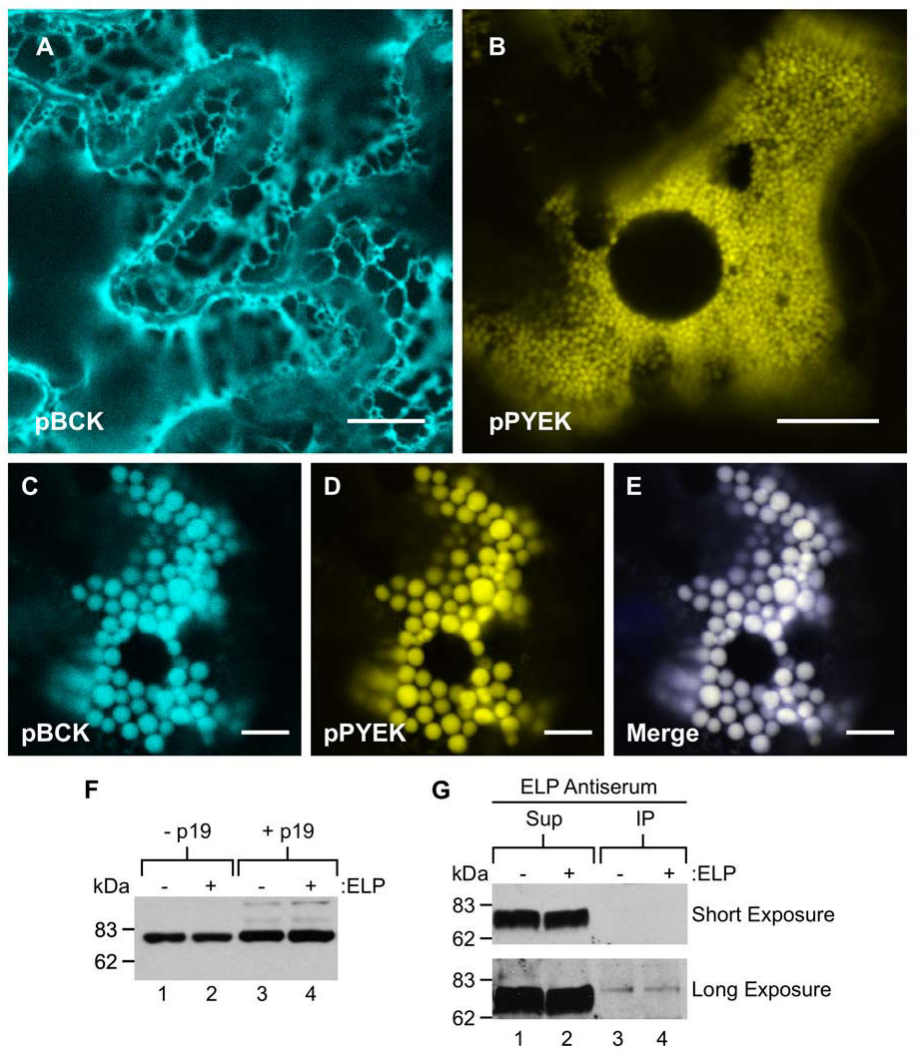
**Endoplasmic reticulum luminal binding protein is localized to the novel protein bodies, but does not specifically interact with elastin-like polypeptide**. **(A) **The tobacco endoplasmic reticulum- (ER-)resident chaperone binding protein (BiP) fused with cyan fluorescent protein (CFP) (pBCK) is appropriately localized to the ER. **(B) **An ER-targeted yellow fluorescent protein-elastin-like polypeptide (YFP-ELP) fusion (pPYEK) accumulated in the induced protein bodies (PBs) located in the cytoplasm. **(C-E) **When co-expressed, pBCK co-localized with the PBs induced by pPYEK expression, suggesting that the novel PBs originate from the ER. **(F) **Western blot analysis comparing BiP accumulation in leaves transiently expressing ER-targeted GFP in the absence (pPGK) or presence (pPGEK) of an ELP tag, with or without co-agro-infiltration of the p19 suppressor of gene silencing. Total protein extracts (30 μg/lane) were resolved by sodium dodecylsulphate-polyacrylamide gel electrophoresis (SDS-PAGE), followed by immunoblotting using an anti-BiP antibody. **(G) **Leaf extracts expressing pPGK or pPGEK were immunoprecipitated with anti-ELP antiserum. The supernatants (Sup) and immunoprecipitates (IP) were separated by SDS-PAGE and probed with anti-BiP antibody. Bar, 10 μm (A, B); 5 μm (C-E).

To provide further validation that the PBs are derived directly from the ER, co-localization of the tobacco ER-resident molecular chaperone binding protein (BiP) fused with CFP (pBCK) and an ER-targeted YFP-ELP fusion (pPYEK) was performed. When expressed alone, pBCK resulted in a fluorescent pattern consistent with an ER-localization (Figure 6A), whereas pPYEK expression resulted in the formation of novel PBs (Figure 6B). When pBCK and pPYEK were transiently co-expressed in *N. benthamiana *leaf epidermal cells along with p19, the BiP-CFP co-localized with the PBs induced by ER-targeted YFP-ELP expression (Figure 6C to 6E). The presence of the ER chaperone BiP within the PB-like structures provides additional support that these accretions originate from the ER.

BiP has been implicated in PB biogenesis [50,51] and has been shown to interact with prolamin-based PBs in a manner that is distinct from its normal chaperone activity [52]. Thus, we examined whether the content of BiP was increased in leaves following the induction of PBs caused by ELP tag expression. Western blotting was performed to compare total BiP protein in crude extracts prepared from pooled samples of infiltrated *N. benthamiana *leaves, expressing ER-targeted GFP (pPGK) and GFP-ELP (pPGEK) in the absence or presence of p19 (Figure 6F). Based on our analysis, the presence of an ELP tag did not affect the levels of BiP in the absence or presence of p19 expression. However, BiP accumulation was increased in the presence of p19 expression, probably caused by the significantly higher accumulation of foreign protein in the ER. These results suggest that the expression of an ELP fusion protein in the ER was not responsible for the induction of BiP.

A computer-based BiP scoring software, developed by Blond-Elguindi *et al*. [53], was used to predict potential BiP-binding motifs within the ELP sequence. BiP preferentially binds heptapeptides containing a high proportion of hydrophobic residues [54]. A single putative BiP binding site (PGVGVPG) with a score of 12 was identified 14 times within the repetitive ELP sequence. Binding motifs with scores greater than 10 have been shown to have an 80% probability of binding to BiP [55]. Consequently, the specific interaction of ELP with BiP was investigated by co-immunoprecipitation analysis. Equal amounts of pooled protein extracts from leaf samples expressing pPGK or pPGEK were immunoprecipitated with anti-ELP antiserum, resolved by SDS-PAGE, blotted onto nitrocellulose and probed with anti-BiP antibody. For both pPGK and pPGEK protein extracts, the vast majority of BiP was found in the supernatant (Figure 6G, lanes 1 and 2), whereas small equivalent amounts of co-immunoselected BiP were only observed with long film exposures (Figure 6G, lanes 3 and 4). This demonstrates that BiP does not specifically associate with the ELP fusion tag, and the small quantity of co-immunoprecipitated BiP detected may be due to its general chaperone-binding properties.

### Subcellular localization of the endoplasmic reticulum-derived novel protein bodies

**Figure 7:**
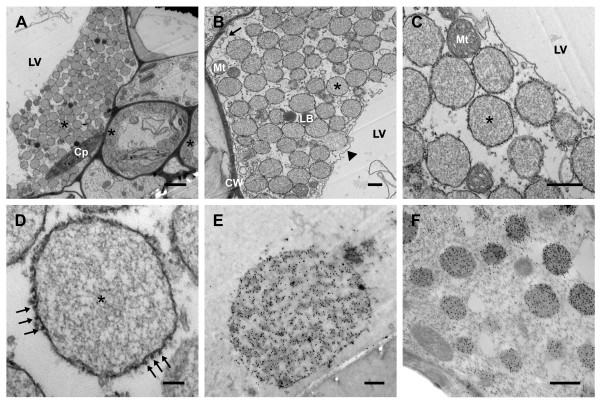
**Subcellular localization of the endoplasmic reticulum-targeted green fluorescent protein-elastin-like polypeptide fusion protein (pPGEK) in *Nicotiana benthamiana *leaves**. **(A) **Electron microscopy confirmed the location of numerous newly-formed endoplasmic reticulum- (ER-)derived protein bodies (PBs) (examples indicated by asterisks) in the cytoplasm of the leaf cells. **(B-D) **Progressively higher magnifications of the PBs seen in (A). (B) The novel PBs occupied the cytosolic space between the tonoplast (indicated by an arrowhead) and the plasma membrane (indicated by an arrow). (C) The PBs were clearly surrounded by a membrane that appears to no longer be contiguous with the ER. (D) The PB membrane was decorated with ribosomes (indicated with arrows), strongly suggesting that the PBs were originally derived from the rough ER. **(E, F) **Immunogold localization confirmed the presence of green fluorescent protein-elastin-like polypeptide (GFP-ELP) inside the novel cytoplasmic PBs in ultrathin sections of *N. benthamiana *leaves using anti-ELP (E) and anti-GFP (F) antibodies detected with goat anti-rabbit or anti-mouse IgG conjugated to 15 nm gold particles. No significant immunolabeling was observed in other subcellular compartments or wild-type plants. The different subcellular compartments were labeled: Cp, chloroplast; CW, cell wall; Mt, mitochondria; LB, lipid body; LV, lytic vacuole; *, induced protein body. Bar, 2 μm (A); 500 nm (B, C, E, F); 100 nm (D).

The addition of an ELP tag to GFP was shown by confocal microscopy to induce the formation of ER-derived PBs; however, we were unable to unequivocally establish whether the PBs remained in the ER or if they were released into the cytosol from the ER. Therefore, electron microscopy (EM) and immunogold labeling were performed on leaf tissue expressing ER-targeted GFP-ELP to further investigate this question. As expected, electron micrographs of the pPGEK-expressing leaf tissues showed the presence of large, electron-dense, spherical PB-like structures in the cells (Figure 7A to 7F). The PBs were typically 0.5 to 1.0 μm in diameter with larger PBs (approximately 4 μm in diameter) also observed at a lower frequency (Figure 7E). The size of the PBs determined by EM was similar to the sizes suggested from the confocal analysis. PB-like structures were not observed in non-transfected leaf sections (data not shown).

Higher magnification provided more detailed images of the PBs, which were clearly surrounded by a distinct membrane that does not appear to be contiguous with the ER network (Figure 7B to 7D), suggesting that the PBs are terminally stored in the cytosol and not retained in the ER. The novel PBs occupy the cytosolic space between the large central lytic vacuole and the cell wall, along with other cytoplasmic organelles such as chloroplasts, mitochondria, and lipid bodies (Figure 7A and 7B). The membrane surrounding the PBs was studded with ribosomes (Figure 7D, arrows), which is strongly suggestive of their derivation from the rough ER [56]. In addition, the PB membranes are likely ER bilayers because monolayer membranes exclude the attachment of ribosome-binding proteins [57].

To further examine the intracellular distribution of ER-targeted GFP-ELP, ultrathin sections of the transfected leaves were immunogold-labeled using antibodies against the ELP and GFP proteins. To optimize for immunoreactivity, the leaf tissue samples were embedded in LR-Gold resin. The novel PBs were strongly labeled with immunogold particles (black dots inside the PBs), targeting either the ELP (Figure 7E) or GFP (Figure 7F) portion of the pPGEK fusion protein. No significant immunolabeling was observed in any other subcellular compartments. Moreover, no background was observed in wild-type leaves or transgenic leaves treated without a primary ELP or GFP antibody (data not shown).

Fluorescence recovery after photobleaching (FRAP) was used to investigate whether the pPGEK protein cycled in and out of the novel PBs. In these experiments, a small region of interest within a single PB [see Movie S9 in Additional file 9] or encompassing many PBs [see Movie S10 in Additional file 10] were selectively photobleached and the fluorescence recovery was monitored over time. After bleaching, most of the GFP fluorescence recovered inside the PBs within 5 min, indicating that continuous movement of pPGEK into the PBs may be occurring from other regions of the cell. Interestingly, we were unable to successfully bleach a specific region within a PB [see Movie S11 in Additional file 11], suggesting that pPGEK protein is very mobile inside the PBs. For all cases examined, the loss and recovery of fluorescence was steady and uniform throughout the volume of the PB.

## Discussion

### The importance of appropriate subcellular targeting for recombinant protein accumulation

Targeting of heterologous proteins to the appropriate subcellular compartment can be critical for obtaining suitable levels of accumulation, since the structure and stability of the recombinant protein is affected by its route and final destination in the cell [58,59]. In addition, proteolytic degradation of the recombinant protein by host proteases can be a major problem [14], along with interferences between the foreign protein and the functions of the host's cellular components [60].

When expressed in the absence of the p19 suppressor of gene silencing, the target protein, GFP, accumulated to the highest level in the cytoplasm, followed by the ER, chloroplasts, and apoplast. The presence of an ELP fusion tag had a negligible effect on the concentration of cytoplasm-, apoplast- or ER-targeted GFP, but significantly decreased the level of chloroplast-targeted GFP. The ELP tag may be preventing translocation across the chloroplast membranes into the stroma. Alternatively, it is possible that the chloroplast-targeted GFP-ELP may be rapidly degraded inside the chloroplasts, thus limiting its ability to accumulate. Previously, ELP was stably produced in tobacco chloroplasts [61], but the yield of ELP was very low compared with other heterologous proteins expressed in genetically engineered chloroplasts [62]. Zeolin, a chimeric protein derived from the maize prolamin γ-zein and the bean vacuolar phaseolin seed storage proteins [15], was also found to accumulate to significantly lower levels in the chloroplasts than in the ER [63]. This was attributed to a decreased stability of Zeolin in the chloroplasts as a result of increased proteolytic activity in this subcellular compartment.

When co-expressed with p19, the presence of an ELP tag significantly increased the concentration of GFP two-fold relative to the control. Under the hyperexpression conditions, ELP's enhancement of recombinant protein accumulation was only observed for the ER-targeted proteins, likely corresponding to increased PB production. Under the same hyperexpression conditions, the cytoplasmic-targeted GFP (pG) and GFP-ELP (pGE) proteins accumulated to similar levels (13.8 ± 2.3% of TSP and 11.1 ± 2.7% of TSP, respectively) and the occurrence of PBs was not detected (data not shown).

### Endoplasmic reticulum-targeted elastin-like polypeptide fusions stimulate the production of a novel type of protein body

To date, ELP tags have been exclusively used as fusion partners to ER-targeted recombinant proteins in plants, since the ER provides the most suitable environment for complex post-translational modifications to occur, such as glycosylation and disulfide bond formation [64]. Moreover, the flexibility of the ER allows for direct accumulation of synthesized proteins in this stable intracellular compartment [65], which has been shown to significantly enhance recombinant protein yields [66-69].

Based on our findings, fusion of an ELP tag to an ER-targeted GFP reporter protein was responsible for significantly enhancing the production of PBs, relative to the unfused control. Although expressed in the leaves of plants, these novel ELP-induced spherical particles appear very similar in size and morphology, based on confocal and electron microscopy analyses, to natural PBs found in the seed of maize [70,71], rice [17,72-74], wheat [3,75], and soybeans [76]. Furthermore, these particles closely resemble the artificially-induced PBs observed in the leaves of tobacco plants when expressing fusions to prolamin seed storage protein derivatives, such as Zeolin [15,16] or Zera [18]. Although prolamin seed storage proteins lack a typical H/KDEL ER-retrieval signal, they possess an unusual ability for being retained in the ER, which has been suggested to play a role in the biogenesis of PBs [5,51,77]. In contrast, the presence of a KDEL motif was necessary for a secreted ELP fusion to induce PB formation in the present study. The KDEL signal may function to concentrate ELP fusion proteins within the ER, resulting in the subsequent formation of novel PBs. Notably, Smith *et al*. [78] demonstrated that expression of the hepatitis B surface antigen in plant cells produced tubular ER-derived structures that accumulate in the cytoplasm.

Presumably, ELP-mediated formation of PBs may protect recombinant proteins from proteolytic degradation. In nature, PBs function to stably accumulate large amounts of storage proteins in seeds. Recently, this process has also been shown to improve heterologous protein accumulation when artificially employed in other plant tissues and other eukaryotic systems [15,18]. Encapsulation into stable intracellular storage organelles may exclude foreign proteins from normal physiological turnover in the plant secretory pathway via ER-associated degradation (ERAD), which is a component of the protein quality control system [18,79,80]. As a result of Zera PB formation, Torrent *et al*. [18] demonstrated that recombinant protein accumulation in leaf material remained stable when dried at 37°C and stored for 5 months at room temperature, which are conditions usually responsible for extensive proteolysis. Furthermore, a transgenic rice seed-based vaccine expressing cholera toxin B subunit was resistant to the harsh environmental conditions of the gastrointestinal tract when administered orally and maintained immunogenicity as a result of its accumulation in stable PBs [81].

All existing evidence suggests that membranous ER-derived PBs are physiologically inert organelles, which can function to segregate recombinant proteins from the host as a means of alleviating any undesirable activities towards each other [18,82]. Therefore, the formation of PBs may provide a promising approach for depositing large amounts of concentrated heterologous proteins within the limited space of the cell, without subjecting the ER to an intolerable level of stress [2,65].

### BiP's role in the formation of elastin-like polypeptide-induced protein bodies

The ER-resident chaperone BiP has been shown to be associated with rice [83] and wheat [75] prolamin PBs. In addition, BiP has been implicated in PB biogenesis because of its role in retaining prolamins in the ER lumen by facilitating their folding and assembly into insoluble PBs in developing seeds [50,51] and transgenic leaves [7,15]. In the present study, BiP was localized to the novel PB-like structures, suggesting that BiP may play a role in the formation of the ELP-induced PBs in transgenic leaves.

Previously, increases in BiP accumulation have been observed in transgenic plants that expressed prolamins and produced PBs [7,84]. Our data indicated that the presence of an ELP fusion tag (pPGEK), which significantly enhanced the formation of PBs, was not capable of inducing BiP accumulation more than the unfused control protein (pPGK). BiP expression correlated with the levels of recombinant protein production, but did not correlate with the levels of induced PBs or the presence or absence of an ELP fusion tag.

Previous studies have demonstrated that specific BiP binding motifs identified within prolamins, such as phaseolin [85] or zein [55], using a BiP scoring software [53], were important for BiP-prolamin protein interactions and the ability to form PBs. Furthermore, BiP has been shown to interact extensively with prolamins in a specific manner, which is unique from its normal chaperone activity [15,52,55]. Although we identified a strong BiP-binding motif repeatedly throughout the ELP sequence, co-immunoprecipitation analysis revealed that no specific interactions exist between ELP and the ER-chaperone BiP, which differentiates ELP-based PBs from prolamin-based PBs. Thus, our studies suggest that BiP is not actively involved in the formation of the ELP-induced PBs and ER resident proteins are just passively incorporated non-specifically into the PBs during their formation.

### Mechanism for elastin-like polypeptide-induced formation of protein bodies: a working model

PBs function in the cells of seeds to store high concentrations of particular proteins in a localized stable organellar environment. In our case, it is possible that ELP enhances the stability and solubility of its respective fusion partners, which increases their levels of accumulation. Thus, ELP-induced PBs may simply form as a result of heterologous protein accumulation reaching a critical local concentration exceeding the normal solubility limit, which subsequently triggers their aggregation and assembly into spherical PBs [86]. This argument is somewhat supported by the fact that ER-targeted unfused GFP was also shown to induce PB formation, albeit to a much lower degree than in the presence of an ELP fusion. To our knowledge, this is the first reported example demonstrating the formation of PBs following expression of ER-targeted GFP, suggesting that findings have to be carefully interpreted when overexpressing foreign proteins. However, overexpression of heterologous proteins is our research objective, as we purposely attempt to maximize recombinant protein production in plants. Aggresomes are often observed in the cytosol of prokaryotes and eukaryotes when overexpressing heterologous proteins [87,88]. However, many examples have shown that accumulation of aggregated proteins in the ER is not sufficient to induce PB formation [65,89,90]. In our case, cytoplasm-targeted GFP and GFP-ELP proteins accumulated to higher levels than their ER-targeted counterparts; however, no aggregation of the heterologous proteins was observed. This suggests that PB formation is specific to ER-related processes. Thus, specific characteristics of the aggregating proteins play a fundamental role in their ability to self-assemble and form discrete PBs [65].

Because of their hydrophobic nature, prolamins have been thought to aggregate in a non-specific manner within the ER lumen, but additional cellular factors are also necessary for their accretion into ER-derived PBs [4,17,91]. Similar to prolamins, ELPs are also relatively rich in hydrophobic amino acids, suggesting that ELPs may aggregate non-specifically with themselves as a means of reducing the hydrophobic effect experienced in the aqueous lumen of the ER [92] by directing the hydrophobic ELP away from the aqueous phase [6]. The intrinsic biophysical properties of seed storage proteins have been shown to be important for the formation of PBs [65]. For example, the PPPVHL repeat domain of Zera, derived from maize γ-zein [18], adopts a spontaneous polyproline II conformation forming an extended amphipathic helix, which is able to self-assemble and form cylindrical micelles [6]. This intramolecular interaction among seed storage proteins appears to be indispensable for their aggregation and the biogenesis of PBs [56,93]. Analogous to seed storage proteins, mammalian-derived ELPs also possess the ability to self-aggregate and undergo co-acervation via an ordered process, leading to the formation of a stable supramolecular structure [31]. As temperature rises, the soluble ELP biopolymer collapses from an extended chain into an insoluble twisted filament structure consisting of β-spirals having type II β-turns [94,95]. The plant growth conditions (that is, temperature) used in this study should not induce the aggregation of an ELP_28 _tag; however, the concentration of ELP is also known to affect its own precipitation behaviour [96,97]. Although not proven, high local concentrations of ELP-fusion proteins in the ER may play a role in their aggregation and subsequent formation into novel PBs.

Prolamin-based PBs generally form directly within the lumen of the ER where they can remain permanently stored, as demonstrated with maize and rice [91]. After formation, the PBs can alternatively bud off from the ER as discrete spherical organelles, where they can either reside in the cytosol as seen for Zera-induced PBs [18] or be sequestered into protein storage vacuoles by autophagy as shown for wheat, barley, and oats [2,4,75]. Based on our confocal and electron microscopy analyses, we hypothesize that ER-targeted ELP fusion proteins are synthesized on ribosomes associated with the rough ER and then transported into the ER lumen, where they accumulate and assemble into PBs by some unknown mechanism. The ER-derived PBs are then thought to disconnect and bud out from the cisternal ER into the cytoplasm where they remain surrounded by ER membranes and are ultimately stored [2,56]. However, the FRAP analysis somewhat challenges the notion of distinct, non-connected, membrane-bound PBs terminally stored in the cytoplasm, since GFP fluorescence recovered in the PBs after they had been photobleached, suggesting continuous cycling of GFP in and out of the PBs. To explain this phenomenon, it is possible that transient or permanent connections may exist between the PBs, which could not be detected given the experimental techniques employed in this study. For example, stromules have been shown to be dynamic structures in plants, enabling transfer of proteins and macromolecules between interconnected plastids [48,98]. Alternatively, membrane transport pathways between the ER and PBs may exist in a similar manner to the specialized close connections that occur between Golgi bodies and the ER [99]. Moreover, the KDEL ER-retrieval motif may be trafficking the soluble protein back to the ER-membrane bound PBs from the Golgi bodies via COPI-mediated retrograde transport [100,101]. Finally, the PBs may subsist as independent protein factories, as we have shown that they are surrounded by ribosome-studded ER membranes and contain ER-resident proteins, including protein-folding chaperones.

The process controlling the size of the ELP-induced PBs remains unknown, but two mechanisms can be proposed. First, variously-sized accretions could form within the ER prior to their release into the cytoplasm. Alternatively, homogeneously-sized individual PBs could bud from the ER once they attain a critical size and subsequently coalesce or fuse with one another in the cytosol to form larger PBs, which has previously been observed for prolamin-based PBs [3,4,57]. Our observations suggest that the latter mechanism is more probable, since the vast majority of PBs had a comparable size, which generally increased with time following agro-infiltration. Because recombinant protein is constantly shunted away from the ER via PBs containing high concentrations of the target protein, the synthesis/degradation equilibrium within the ER will shift towards increased synthesis without excessive build-up becoming fatally toxic to the cell. Thus, a continual renewal of ER-production capacity may exist, which may be responsible for allowing subsequently higher levels of heterologous protein to accumulate inside the cell.

Our results, along with others, indicate that seed-specific factors are not required for the formation of PBs in vegetative leaf tissues [5,15,17,56], mammalian cell cultures, insect cells, and fungal cultures [18]. In fact, given that expression of mammalian-derived ELP was sufficient to induce the formation of PB-like organelles in plants, seed storage proteins may not be required in this process either. These findings suggest that the ER possesses an intrinsic ability to form PB-like accretions in eukaryotic cells when overexpressing particular proteins with special physicochemical properties, such as ELP.

## Conclusion

This study's main objectives were to determine the utility of ELP in various subcellular compartments and to elucidate ELP's mechanism of action for increasing recombinant protein accumulation in the ER of plants. In summary, the presence of an ELP fusion tag had a negligible effect on the concentration of GFP in the cytoplasm and apoplast, whereas it decreased the accumulation of GFP in the chloroplasts. On the other hand, ELP was shown to significantly enhance the yield of GFP to 11% of TSP when hyperexpressed in the ER, in the presence of the p19 suppressor of gene silencing. Based on confocal and electron microscopy analyses, our findings indicated that ELP fusions targeted to the ER induced the formation of novel PB-like structures in leaves, which may exclude the heterologous protein from normal physiological turnover and may be responsible for ELP's positive effect on recombinant protein accumulation. Interestingly, the ELP-induced PBs were highly mobile organelles, exhibiting various dynamic patterns of movement throughout the cells, which were dependent on intact actin microfilaments and a functional actomyosin motility system. Further studies indicated that the novel PBs were derived from the ER and were terminally stored in the cytoplasm, since they: (i) contained proteins with ER-specific glycans; (ii) contained an ER-resident BiP protein; and (iii) were surrounded by a distinct membrane studded with ribosomes. In addition, our results indicate that BiP does not specifically associate with the ELP tag and is not actively involved in the formation of the ELP-induced PBs. PB formation enables high local concentrations of heterologous proteins to exist within the limited space of the cell, while insulating the protein from normal cellular protein degradation mechanisms, and without subjecting the ER to an intolerable level of stress [18,65]. Therefore, an ER-targeted ELP-fusion approach provides an effective strategy for enhancing the production yield of recombinant proteins in plant leaves via accumulation in stable PB-like organelles.

## Methods

### Construction of plant expression vectors

The coding sequences of GFP, ELP (28 × VPGVG) and tobacco ER luminal BiP, along with their additional 5' and 3' tags, were constructed using a combined ligase chain reaction/polymerase chain reaction (LCR/PCR) approach [102]. This technique utilizes a set of overlapping oligonucleotides designed by the Web-based program Gene2Oligo [103] to assemble synthetic genes. The coding sequences of yellow (YFP) and cyan (CFP) fluorescent proteins were PCR-amplified from plasmids kindly provided by Federica Brandizzi (Michigan State University, USA). The Pr1b secretory signal peptide from tobacco [104] was fused in-frame to the YFP and CFP genetic sequence by homology overlap PCR. A *Kas*I restriction site was added to the 3'-end of GFP, YFP and CFP to create a two-amino-acid linker (glycine-alanine) and to allow for in-frame ligation with the C-terminal ELP fusion tag. An ER retrieval signal (KDEL) was added to the C-terminus of certain constructs by extension PCR. To assist in subsequent cloning steps, *BamH*I and *EcoR*I restriction sites were incorporated at the 5'- and 3'-end of all completed constructs. The final constructs were moved into the plant binary expression vector pCaMterX, where the coding sequences were placed under the control of the dual-enhancer cauliflower mosaic virus (CaMV) 35S promoter [105], a tCUP translational enhancer [106], and the nopaline synthase (nos) terminator. The expression constructs were electroporated into *Agrobacterium tumefaciens *strain EHA105 [107] and then used for plant transformation. Constructs for expressing the mouse talin (mTalin)-YFP fusion protein and the tail of myosin XI-K were kindly provided by Aiming Wang (Agriculture & Agri-Food Canada, Canada) and Valerian Dolja (Oregon State University, USA), respectively.

### Agro-infiltration of plant leaves

For transient expression, the intact leaves of 6 to 8-week old *N. benthamiana *plants were infiltrated with *Agrobacterium *strains as previously described [108,109]. Briefly, the induced *Agrobacterium *suspensions were adjusted to a final optical density at 600 nm (OD_600_) of 1.0 and then directly injected into the intercellular spaces of leaves using a 1-ml syringe with a 29-gauge needle. For the co-infiltrations, the bacterial strains were each adjusted to an OD_600 _of 1.0, prior to being mixed together in equal amounts. After infiltration, the plants were maintained for 3 to 6 days in a controlled growth chamber at 22°C with a 16-h photoperiod. For the quantitative fluorometric analysis, each biological replicate was represented by an agro-infiltrated leaf panel, where a panel is the area between the midrib and secondary veins. To compensate for variability between plants, leaves and location on a leaf, comparably-sized leaves from eight different plants of similar age were systematically agro-infiltrated for each expression construct. The individually infiltrated panels were sampled 4 days post-transfection and analyzed separately by fluorometry.

### Quantification of green fluorescent protein levels

For each leaf sample, the TSP was extracted and the concentration of GFP was determined by quantitative fluorometric analysis as described by Conley *et al*. [25].

### Deglycosylation, co-immunoprecipitation, and Western blot analysis

Total plant protein extracts were deglycosylated with PNGaseF (New England Biolabs, Ipswich, MA, USA) or EndoH (Sigma, St Louis, MO, USA) for 24 h at 37°C, according to the manufacturer's instructions. PNGaseF cleaves all high-mannose, hybrid, and complex-type oligosaccharides from *N*-linked glycoproteins, except for those glycans containing a core α(1,3)-linked fucose residue. EndoH is able to remove high-mannose *N*-linked glycans, but not complex-type glycans, from glycoproteins. Control samples were treated the same, except that no enzyme was added. Proteins were co-immunoprecipitated with anti-ELP rabbit serum [28] from plant extracts using a Protein G Immunoprecipitation Kit (IP50, Sigma) according to the manufacturer's instructions. The samples were analyzed by sodium dodecylsulphate-polyacrylamide gel electrophoresis (SDS-PAGE) and immunoblotted according to Conley *et al*. [66]. The membranes were incubated with a 1:500 dilution of mouse anti-GFP antibody (11814460001; Roche, Mannheim, Germany) or a 1:500 dilution of mouse anti-BiP antibody (SPA818; Stressgen, Michigan, USA) and the primary antibody was detected with a 1:5000 dilution of HRP-conjugated goat anti-mouse IgG (170-6516; Bio-Rad, Hercules, CA, USA).

### Confocal microscopy

A Leica TCS SP2 confocal laser scanning microscope (Leica Microsystems, Wetzlar, Germany) equipped with a 63× water immersion objective was used to examine the subcellular localization of GFP, CFP, YFP, and chlorophyll fluorescence. For the time-lapse experiments, the consecutive images were taken with a Zeiss LSM5 Duo Vario2 confocal microscope (Carl Zeiss AG, Oberkochen, Germany) at the Biotron Imaging Facility (University of Western Ontario, London, Canada). For the imaging of GFP expression and chlorophyll autofluorescence, excitation with a 488 nm argon laser was used and fluorescence was detected at 500 to 525 nm and 630 to 690 nm, respectively. The excitation wavelength for CFP was 405 nm and its emission was recorded at 440 to 485 nm. For visualization of YFP, excitation at 514 nm was used along with an emission window set at 520 to 550 nm. For the CFP and YFP co-localization experiments, simultaneous imaging was conducted using the line-sequential multitrack scanning mode of the microscope to exclude the possibility of crosstalk between the fluorophores.

### Latrunculin B treatment

For actin depolymerization, a 25 μM solution of latrunculin B (Sigma) was injected into the abaxial surface of the leaf, and the resulting tissue was excised and mounted in latrunculin B solution. Drug treatment was performed for 1 h. The working solution of latrunculin B was prepared fresh from a frozen stock solution (10 mM in DMSO).

### Electron microscopy

Small pieces of agro-infiltrated leaves, sampled 4 days post-infiltration, were fixed overnight at room temperature (RT) by vacuum infiltration with 4% paraformaldehyde and 0.5% glutaraldehyde in 100 mM phosphate buffer, pH 7.2. The tissue samples were washed three times with phosphate buffer and then post-fixed with 2% osmium tetroxide overnight at RT. After three washes with the buffer, samples were dehydrated through an acetone series and embedded in a mixture of EPON 812 (17%), Araldite 502 (13%), and dodecenyl succinic anhydride (67%) containing 3% DMP-30 as an accelerator. After curing for 48 h at 60°C, ultrathin tissue sections were cut and mounted onto nickel grids. The grids were stained with 5% uranyl acetate for 10 min followed by a 1 min staining with saturated lead citrate and examined with a transmission electron microscope (CM10; Philips, Eindhoven, the Netherlands).

### Immunogold labeling

For immunolabeling of the leaf tissue, samples were fixed as described above, but the secondary fixative step was omitted. The tissue samples were dehydrated through an ethanol series and embedded with LR-Gold (London Resin Company Ltd, London, UK), as described previously [110]. The nickel grids containing the ultrathin tissue sections were first incubated in Goat Blocking Solution (Aurion, Wageningen, the Netherlands) for 30 min at RT. The grids were then incubated for 2 h at RT with an anti-ELP rabbit serum (1/100) or anti-GFP mAb (632380; BD Biosciences, Mississauga, Canada, 1/10) resuspended in dilution buffer (PBS, 0.05% Tween-20, 0.2% BSA-c (Aurion), pH 7.2). As controls, similar samples were incubated with pre-immune rabbit serum (for anti-ELP serum) or dilution buffer (for anti-GFP mAb). After three washes with dilution buffer, grids were incubated for 1 h with goat anti-rabbit or anti-mouse secondary antibodies conjugated to 15 nm gold particles, followed by three more washes with dilution buffer and three washes with distilled water. The grids were stained as described above and examined under a transmission electron microscope.

### Statistical analysis

The statistical analysis was performed with SPSS 16.0 for Windows. The normal distribution of the data was confirmed with the Lilliefors's test. One-way ANOVA followed by pairwise comparisons with the Tamhane's T2 test was used to analyze the data presented in Figure 3. Figure 4A data was analyzed with the Student's t-test. For all tests, the level of statistical significance was defined as *P *< 0.05.

## Abbreviations

BiP: endoplasmic reticulum luminal binding protein; CFP: cyan fluorescent protein; ELP: elastin-like polypeptide; EM: electron microscopy; ER: endoplasmic reticulum; ERAD: ER-associated degradation; FRAP: fluorescence recovery after photobleaching; GFP: green fluorescent protein; ITC: inverse transition cycling; LCR/PCR: ligase chain reaction/polymerase chain reaction; PB: protein body; RT: room temperature; TSP: total soluble protein; YFP: yellow fluorescent protein.

## Authors' contributions

AJC and JEB conceived and designed the study and JJJ and RM participated in its design. AJC performed all experiments except for the immunoelectron microscopy analysis, which was carried out by JJJ. AJC analyzed the data and wrote the manuscript. JJJ performed the statistical analysis. JEB and RM supervised the work. All authors have read and approved the final manuscript.

## Supplementary Material

Additional file 1 **Movie S1. A three-dimensional rendering of a cluster of novel protein bodies**. Sixty confocal images of a *Nicotiana benthamiana *epidermal cell expressing an endoplasmic reticulum- (ER-)targeted green fluorescent protein-elastin-like polypeptide (GFP-ELP) fusion protein (pPGEK) were taken from a 6.00 μm projection in the Z-direction and compiled together to construct the rotating 3-D image representing a collection of protein bodies. Click here for file

Additional file 2 **Movie S2. The variously-sized protein bodies are densely packed throughout the cytoplasm of the cell**. Consecutive confocal images were taken and assembled together in a time-lapse movie as the confocal plane progressed through the PGEK-expressing cell. One hundred and ten image frames were taken through an 11.00 μm projection in the Z-direction. Click here for file

Additional file 3 **Movie S3. The protein bodies compactly gather in the cytoplasmic space surrounding the cell's nucleus**. Time-lapse confocal imaging as the confocal plane progressed through the cell expressing pPGEK. Seventy image frames were taken through a 14.00 μm projection in the Z-direction. Click here for file

Additional file 4 **Movie S4. Movement of the protein bodies within *Nicotiana benthamiana *leaf epidermal cells**. Time-lapse confocal imaging of cells expressing endoplasmic reticulum- (ER-)targeted green fluorescent protein-elastin-like polypeptide (pPGEK) was performed to demonstrate the mobility of the novel protein bodies (PBs). A variety of patterns of movement were observed for the PBs. The PBs did not generally move with a constant velocity; rather, they sporadically moved about in a stop-and-go, saltatory fashion. Trafficking of the PBs appeared to occur along the underlying cortical ER network in the leaf cells. Sixty image frames were taken over the course of 2 min 58 s. Click here for file

Additional file 5 **Movie S5. Directed trafficking of protein bodies within the cell**. Consecutive confocal images of PGEK-expressing cells were taken to show that some protein bodies (PBs) remained relatively still and slowly oscillated in position for an extended period resembling Brownian motion, while other resting PBs would suddenly accelerate. Observing the center region of the movie demonstrated that large numbers of PBs moved in a relatively constant direction with a steady velocity towards a specific area of the cell, until moving out of the confocal plane. Two hundred image frames were taken over the course of 8 min 15 s. Click here for file

Additional file 6 **Movie S6. Mobility of novel protein bodies in numerous neighboring cells expressing pPGEK**. The speed, amount and type of protein body (PB) movement is highly variable between neighboring cells. Moreover, particular PBs were observed to be carried away at high velocities when they jumped onto streaming cytoplasmic strands. One hundred and fifty image frames were taken over the course of 6 min 11 s. Click here for file

Additional file 7 **Movie S7. The trafficking of protein bodies is dependent on intact microfilaments**. A 25-μM solution of latrunculin B, a drug responsible for inducing disintegration of the actin cytoskeleton, was infiltrated into the abaxial surface of *Nicotiana benthamiana *leaves that were transiently expressing pPGEK. After 1 h of treatment, the infiltrated area was observed by time-lapse imaging via confocal microscopy. Depolymerization of the cytoskeleton prevented all translational movement of the induced protein bodies (PBs), demonstrating that intact microfilaments are necessary for normal PB trafficking. Fifty image frames were taken over the course of 2 min 37 s. Click here for file

Additional file 8 **Movie S8. Overexpression of a myosin tail inhibits movement of the novel protein bodies**. A dominant-negative mutant of myosin XI-K was co-agro-infiltrated with pPGEK into the leaves of *Nicotiana benthamiana *and visualized 3 days post-transfection by confocal microscopy. As a result, protein body (PB) trafficking was prevented with the PBs simply oscillating in place, suggesting that a functional actomyosin motility system is required for active PB movement, but is not necessary for the formation of PBs. Fifty image frames were taken over the course of 3 min 34 s. Click here for file

Additional file 9 **Movie S9. Fluorescence recovery after photobleaching analysis of an endoplasmic reticulum-targeted green fluorescent protein-elastin-like polypeptide fusion protein (pPGEK) present within the novel protein bodies**. A small region of interest (white circle) within a single protein body (PB) was photobleached. After bleaching, fluorescence recovered in the PB. Eighty six image frames were taken over the course of 3 min 3 s. Click here for file

Additional file 10 **Movie S10. Movement of pPGEK protein into protein bodies following photobleaching**. Selective photobleaching of six protein bodies in close proximity (white circle) showing fluorescence recovery of pPGEK inside the bleached area. One hundred and seventeen image frames were taken over the course of 4 min 15 s. Click here for file

Additional file 11 **Movie S11. The free exchange of proteins occurs rapidly within protein bodies**. A particularly large protein body (PB) was continuously bleached (white circle) and imaged in an alternating fashion for a relatively long period of time. Although only a small region of the PB was selectively photobleached, the entire volume of the PB was homogeneously bleached with time, suggesting very rapid mobility of proteins within the confines of the PB. Twenty eight image frames were taken over the course of 2 min 18 s. Click here for file
